# Expansion of CD56^dim^CD16^neg^ NK Cell Subset and Increased Inhibitory KIRs in Hospitalized COVID-19 Patients

**DOI:** 10.3390/v14010046

**Published:** 2021-12-28

**Authors:** José L. Casado, Elisa Moraga, Pilar Vizcarra, Héctor Velasco, Adrián Martín-Hondarza, Johannes Haemmerle, Sandra Gómez, Carmen Quereda, Alejandro Vallejo

**Affiliations:** 1 Department of Infectious Diseases, Ramón y Cajal Institute for Health Research (IRyCIS), University Hospital Ramón y Cajal, 28034 Madrid, Spain; elisa.moraga.lopez@gmail.com (E.M.); pilar1vizcarra@gmail.com (P.V.); segovia.hve@gmail.com (H.V.); adrian-m@hotmail.com (A.M.-H.); sandra.gomez.maldonado@hotmail.com (S.G.); cqueredar.hrc@salud.madrid.org (C.Q.); 2 Laboratory of Immunovirology, Ramón y Cajal Institute for Health Research, University Hospital Ramón y Cajal, 28034 Madrid, Spain; 3 Department of Prevention of Occupational Risks, University Hospital Ramón y Cajal, 28034 Madrid, Spain; johannes.a.haemmerle@gmail.com

**Keywords:** SARS-CoV-2, COVID-19, NK cell subsets, KIR receptors

## Abstract

Severe Acute Respiratory Syndrome Coronavirus (SARS-CoV-2) infection induces elevated levels of inflammatory cytokines, which are mainly produced by the innate response to the virus. The role of NK cells, which are potent producers of IFN-γ and cytotoxicity, has not been sufficiently studied in the setting of SARS-CoV-2 infection. We confirmed a different distribution of NK cell subsets in hospitalized COVID-19 patients despite their NK cell deficiency. The impairment of this innate defense is mainly focused on the cytotoxic capacity of the CD56^dim^ NK cells. On the one hand, we found an expansion of the CD56^dim^CD16^neg^ NK subset, lower cytotoxic capacities, and high frequencies of inhibitory 2DL1 and 2DL1/S1 KIR receptors in COVID-19 patients. On the other hand, the depletion of CD56^dim^CD16^dim/bright^ NK cell subsets, high cytotoxic capacities, and high frequencies of inhibitory 2DL1 KIR receptors were found in COVID-19 patients. In contrast, no differences in the distribution of CD56^bright^ NK cell subsets were found in this study. These alterations in the distribution and phenotype of NK cells might enhance the impairment of this crucial innate line of defense during COVID-19 infection.

## 1. Introduction

Severe acute respiratory syndrome coronavirus 2 (SARS-CoV-2) infection has continued as a worldwide pandemic since December 2019 [1,2]. More than 200 million confirmed cases and more than 4 million deaths have been reported globally as of October 2021 [3]. Most infected patients recover spontaneously with mild symptoms, but others develop symptoms that require hospitalization, including acute respiratory distress syndrome, multiorgan failure, and death [4]. Immune responses to this disease have been extensively studied, but the role of innate immunity remains unclear [5,6].

The major subset of innate lymphocytes that plays an important role in early protection against viruses is natural killer (NK) cells [7,8,9]. These are also important in the regulation of the humoral and cellular adaptive immune responses. NK cells can be subdivided into subsets based on their relative surface expression of CD56 and CD16 receptors: the CD56^dim^CD16^pos^ cell subset is considered to be the cytolytic cell subset, the CD56^bright^CD16^neg^ cell subset includes the cytokine-producing cells, and the unconventional CD56^dim^CD16^neg^ cell subset has been reported to expand in several clinical conditions [10,11,12].

It has been reported that NK cells in patients with severe COVID-19 are depleted and functionally impaired; show activated phenotypes, proliferation markers, and effector function gene signatures; and present exhaustion phenotypes [13,14].

The CD56^dim^CD16^pos^ NK cell subset with high a KIR expression represents most of the lymphocytes present in the lungs [15]. This cell subset includes the memory-like NK cells that seem to play an important role in the fight against SARS-CoV-2 infection, modulating the immune response through their KIR receptors [16,17].

In the present study, we analyzed the phenotypic distribution of NK cell subpopulations and the expression of activating and inhibitory KIRs in hospitalized COVID-19 patients compared to convalescent and infection-naïve individuals.

## 2. Materials and Methods

### 2.1. Study Design, Participants and Methods

This was a retrospective cross-sectional study comparing three cohorts of individuals. Cohorts included COVID-19 patients hospitalized in the University Hospital Ramón y Cajal, Madrid, Spain, during the first wave of the pandemic (April to May 2020). These patients were diagnosed an average of 8.2 days from the onset of symptoms by the detection of SARS-CoV-2 RNA in patients’ nasopharyngeal swabs using real-time RT-PCR and specific IgA, IgM, and IgG antibodies (COVID-19 IgG/IgM Rapid Test Kit, UNscience Biotechnology, Wuhan, China; COVID-19-SARS-CoV-2 IgA ELISA, Demeditech, Germany). We also collected samples from health care workers (HCW) of the same hospital who had asymptomatic or mild COVID-19 who did not need hospitalization. These were diagnosed by serology and real-time RT-PCR (in individuals with symptoms). Seronegative HCWs without any compatible COVID-19 symptoms were included as infection-naïve individuals. Samples from HCWs were collected from May to June 2020.

EDTA blood was collected from all of the subjects. Peripheral blood mononuclear cells (PBMC) were isolated by Ficoll–Paque density gradient centrifugation using a lymphocyte separation medium (Corning, New York, NY, USA) and cryopreserved until use. 

All individuals included in the study provided either oral or written informed consent. This study was conducted following the Declaration of Helsinki (1996) and approved by our Hospital Ethics Review Committee (EC162/20).

### 2.2. Cytokine Quantification

Plasma were used for the quantification of IFN-γ, IL6 cytokines (MACSPlex Cytokine kit, Miltenyi, Bergisch Gladbach, Germany), and C-reactive protein (CRP Human ELISA kit, Thermofisher Scientific, Waltham, MA, USA) according to the manufacturer’s instructions. 

### 2.3. NK Cell Immunophenotyping

**Figure 1:**
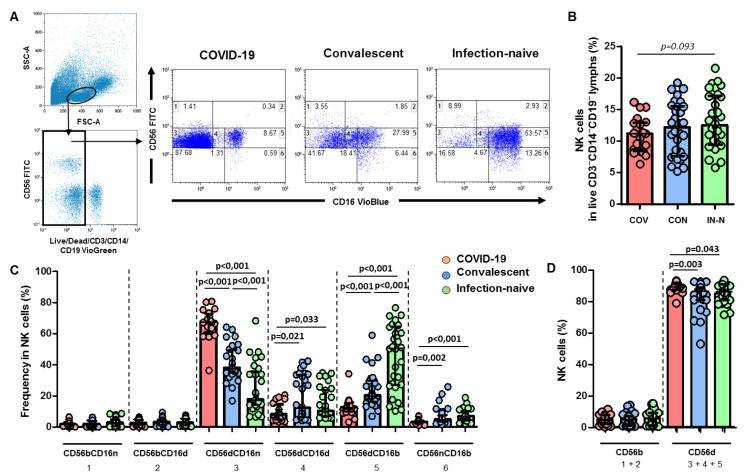
NK cell subset distribution and phenotypes using flow cytometry. Data from samples obtained from COVID-19 patients, convalescent, and infection-naïve individuals are presented. (**A**) Representative flow cytometry plots showing the gating strategy for NK cell phenotyping in CD3^neg^CD14^neg^CD19^neg^ lymphocytes using CD56 and CD16 antibodies. The percentage of each NK cell subset is shown. (**B**) Bar plot showing cumulative data regarding the frequency of total NK cells in COVID-19 patients (red, *n* = 20), convalescents (blue, *n* = 30), and infection-naïve (green, *n* = 30) individuals. One dot represents one patient. (**C**) Bar plots showing cumulative data regarding the relative frequencies of each subset among total NK cells in COVID-19 patients (red), convalescents (blue), and infection-naïve (green) individuals with the identification of each NK cell subset: subset 1 represents CD56^bright^CD16^neg^ NK cells, subset 2 presents CD56^bright^CD16^dim^ NK cells, subset 3 presents CD56^dim^CD16^neg^ NK cells, subset 4 presents CD56^dim^CD16^dim^ NK cells, subset 5 presents CD56^dim^CD16^bright^ cells, and subset 6 presents CD56^neg^CD16^bright^ cells. The relative frequencies of each subset among NK cells are presented. (**D**) Bar plot showing cumulative data regarding the frequency of CD56^bright^ (subsets 1 and 2) and CD56^dim^ (subsets 3, 4, and 5) in COVID-19 patients (red), convalescents (blue), and infection-naïve (green) individuals. FSC-A, forward scatter-area; SSC-A, side scatter-area; statistically significant values are showed in bold when *p* < 0.05 and italic when *p* < 0.1.

For NK cell flow cytometry, cryopreserved PBMCs were thawed and stained for surface protein staining using the viability fixable dye (Viability 405/520 Fixable Dye, Miltenyi, Bergisch Gladbach, Germany) for 5 min at room temperature in the dark. Then, the cells were stained with multiple fluorochrome-conjugated antibodies against surface proteins for 20 min at 4 °C and washed with PBS. Antibodies used for the analysis included CD3-VioGreen, CD14-ViGreen, CD19-VioGreen, CD56-FITC, CD16-VioBlue, 2DL5-PE Vio770, 2DL4-PE, 3DL1-APC-Vio770, 2DS4-APC, 3DL1/L2-PE-Vio770, 2DL2/L3-PE, 2DL1/S1-APC-Vio770, and 2DL1-APC (Miltenyi, Bergisch Gladbach, Germany). For the analysis of NK cell subpopulations from PBMCs, the gating strategies are presented in Figure 1A. Briefly, after singlet cells were gated (FSS-A/FSS-H dot plot), positive cells for live/dead, anti-CD14, anti-CD19, and anti-CD3 were excluded. CD56^+^ and CD16^+^ cells were gated as NK cells and analyzed for conventional CD56^bright^CD16^neg^ (cCD56^bright^), conventional CD56^dim^CD16^pos^ (cCD56^dim^), and uCD56^dim^ NK cells. The expression of KIR receptors within each NK subpopulation was also analyzed. Flow cytometry was performed using a MACSQuant 10 instrument and the data were analyzed using the MACSQuantify software (Miltenyi, Bergisch Gladbach, Germany).

### 2.4. Statistical Analysis

Continuous variables were expressed as medians and interquartile ranges (IQ25–75) and categorical variables by frequencies and proportions. The Mann–Whitney U test (non-parametric) for independent samples was used to compare continuous variables. The nonparametric Spearman rank correlation test was used to evaluate correlations between two parameters. The 2-way ANOVA test was used in group comparisons. Differences between categorical variables were evaluated using contingency tables (Chi-square distribution). Two-sided test was used for all statistics and *p* < 0.05 was considered statistically significant. Data analysis was performed using the Statistical Package for Social Sciences software (SPSS Version 22.0, Chicago, IL, USA). Figures were created using the Prism software version 8.4.3 (GraphPad Software, San Diego, CA, USA).

## 3. Results

A total of 80 individuals, including 20 hospitalized patients (with no need for intensive care unit admission) with COVID-19 (SOFA score ≤ 3), 30 convalescent individuals who were not hospitalized, and 30 infection-naïve individuals, were included in this study. The clinical characteristics of the participants are shown in Table 1. Hospitalized COVID-19 patients were older (*p* < 0.001), were mostly male (*p* < 0.001), and had elevated BMI scores (*p* = 0.008) and comorbidities (*p* = 0.015) compared to the convalescent and infection-naïve individuals (ANOVA analysis).

We analyzed the phenotype of the NK cells among singlet live CD3^neg^CD14^neg^CD19^neg^ lymphocytes using multiparametric flow cytometry analysis (gating strategy shown in Figure 1A). A slightly lower level of total NK cells in lymphocytes was found in COVID-19 patients compared to infection-naïve individuals (*p* = 0.093; Figure 1B).

### 3.1. High Levels of CD56^bright^CD16^neg^ NK Cell Subset Positive for KIR2DS4 Receptor in Hospitalized COVID-19 Patients

**Figure 2:**
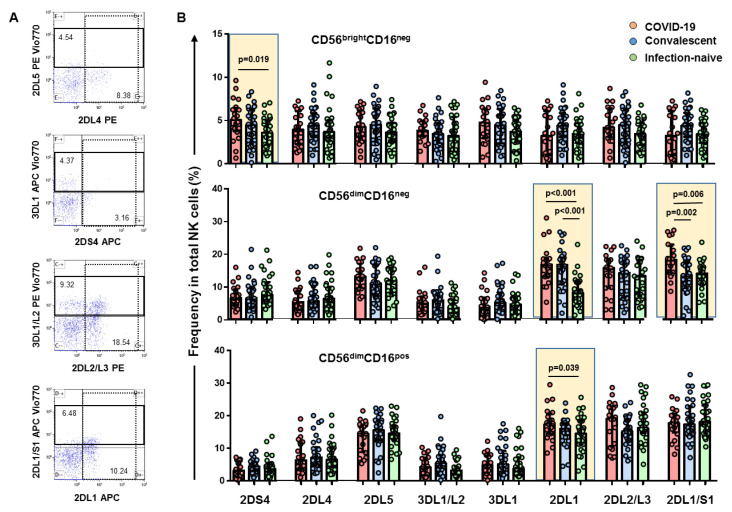
Frequency of NK cell subsets positive for KIR receptors. (**A**), Representative flow cytometry plots of one infection-naïve individual showing the gating strategy for KIR receptors: 2DS4, 2 DL4, 2DL5, 3DL1/L2, 3DL1, 2DL1, 2DL2/L3, and 2DL1/S1 in CD56dimCD16neg NK cell subset showing the percentage of cells for each KIR. (**B**), Bar plot showing cumulative data regarding the frequency of CD56^bright^CD16^neg^, CD56^dim^CD16^neg^, and CD56^dim^CD16^pos^ NK cell subsets for the different KIR receptors in COVID-19 patients (red, *n* = 20), convalescents (blue, *n* = 30), and infection-naïve (green, *n* = 30) individuals. Only statistically significant values are shown in bold when *p* < 0.05.

**Figure 3:**
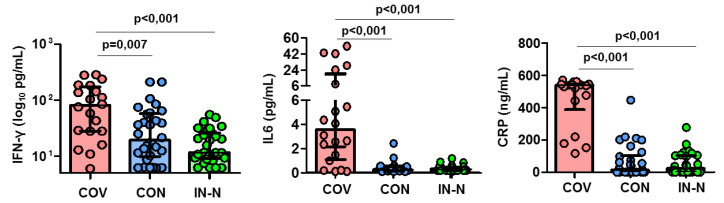
Plasma levels of IFN-γ, IL6 and CRP in COVID-19 patients (red, *n* = 20), convalescents (blue, *n* = 30), and infection-naïve (green, *n* = 30) individuals. Significant when *p* < 0.05.

The frequency of the CD56^bright^CD16^neg^ NK cells did not differ in the three groups studied, as shown in Figure 1C,D. Nevertheless, a higher expression of the activating 2DS4 receptor was found on CD56^bright^CD16^neg^ NK cells in COVID-19 patients compared to infection-naïve individuals (*p* = 0.019), as shown in Figure 2B (see Figure 2A for flow cytometry strategy for KIR receptors). We also found elevated plasma levels of IFN-γ, IL6, and C-reactive protein in samples from COVID-19 patients (Figure 3).

### 3.2. Expansion of CD56^dim^CD16^neg^ NK Cell Subset and Higher Frequency of KIR2DL1 and KIR2DL1/S1 Inhibitor Receptors in Hospitalized COVID-19 Patients

The frequency of the CD56^dim^CD16^neg^ NK cell subset was significantly higher compared to that of either convalescents (*p* < 0.001) or infection-naïve (*p* < 0.001) individuals (Figure 1C). The CD56^dim^CD16^neg^ cell subset expressing the 2DL1 and 2DL1/S1 receptors was higher in COVID-19 patients compared to both convalescent (*p* < 0.001 and *p* = 0.002, respectively) and infection-naïve individuals (*p* < 0.001 and *p* = 0.006, respectively), as shown in Figure 2B.

### 3.3. Depletion of CD56^dim^CD16^dim^ and CD56dimCD16bright NK Cell Subsets with a Higher Frequency of KIR2DL1 Inhibitory Receptor

The frequency of both CD56^dim^CD16^dim^ and CD56^dim^CD16^bright^ NK cell subsets was lower compared to that of convalescent (*p* = 0.021 and *p* < 0.001, respectively) and infection-naïve individuals (*p* = 0.033 and *p* < 0.001, respectively), as shown in Figure 1C. In parallel, the frequency of CD56^dim^CD16^dim/bright^ NK cells positive for 2DL1 inhibitory receptors was higher in hospitalized COVID-19 patients compared to infection-naïve individuals (*p* = 0.039), as shown in Figure 2B.

### 3.4. NKs Cell Subset and KIRs Associations to Inflammatory Markers

**Figure 4:**
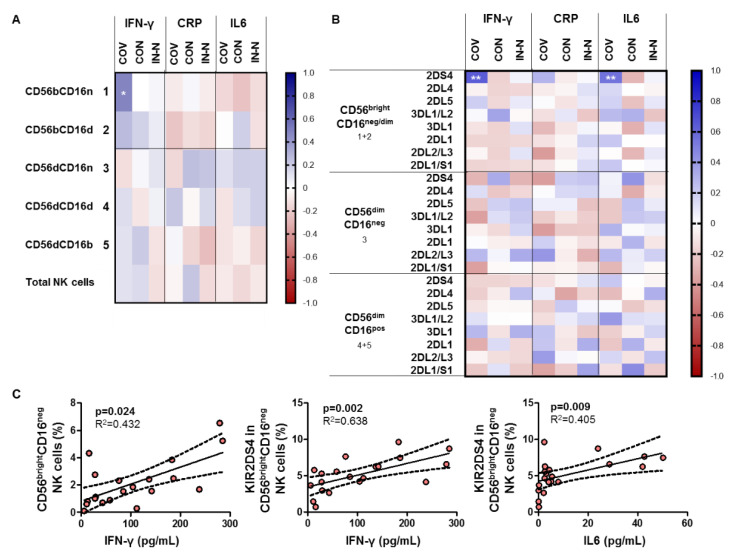
Association of NK cell subsets and KIRs with inflammatory markers. Heatmap graphs representing correlations between the percentages of NK cell subsets (**A**) and KIR receptors (**B**) with inflammatory marker levels in COVID-19 patients, convalescents, and infection-naïve individuals. The blue color represents a positive correlation and the red color represents a negative correlation. The intensity of the color indicates the R^2^ coefficient. (**C**), correlation graphs for those correlations that are significant in A and B. Statistical significance data are highlighted with white squares. * *p* < 0.05; ** *p* < 0.01. Spearman test was used for nonparametric correlations.

Correlations between NK cell subsets and KIRs are shown in Figure 4 as a heatmap graph. No correlation was found between the total NK cells or NK cell subset and inflammation markers in COVID-19 patients, convalescents, or infection-naïve individuals (Figure 4A). On the other hand, a significant positive correlation between KIR 2DS4 expressed in the CD56brightCD16neg/dim cell subset and both the IFN-γ and IL6 levels (*p* = 0.002 and *p* = 0.008, respectively) was found in COVID-19 patients (Figure 4B).

## 4. Discussion

The role of NK cell subtypes and the killer immunoglobulin-like receptors (KIRs) that regulate their function during SARS-CoV-2 infection is still under investigation, since research has been mainly focused on the adaptive immune responses. Impaired NK cell counts and functionality [18], as well as deficits in other members of the innate immunity such as dendritic cells that include alterations in their homing and activation markers in SARS-COV-2-infected patients [19], have been reported. In the present study, we aimed to characterize the phenotypic and KIR expression changes in NK cells during COVID-19. Our results showed an overall reduction in total NK cells and an expansion of the CD56^dim^CD16^neg^ NK cell subset in parallel with the depletion of the CD56^dim^CD16^dim/bright^ NK cell subsets, in line with other reports [20,21,22,23]. Interestingly, the expansion of the CD56^dim^CD16^neg^ NK cell subset was concomitant with the expansion of this subset positive for inhibitory KIR receptors 2DL1 and 2DL1/S1 in hospitalized COVID-19 patients. On the other hand, a reduction in the frequency of either CD56^dim^CD16^dim^ or CD56^dim^CD16^bright^ in parallel with a higher frequency of these cell subsets positive for inhibitory 2DL1 receptor reinforced the impaired situation of this innate NK cell defense line. Moreover, although COVID-19 patients showed similar frequencies of CD56^bright^CD16^neg^ NK cells compared to convalescent and infection-naïve individuals, higher levels of positive cells for activating 2DS4 receptor were found in COVID-19 patients. This NK cell subset was reported to express reduced levels of KIR receptors and mainly produce cytokines upon infection [20,24]. Of note, neither gender nor age was associated with the different distribution of NK cell subsets or the expression of KIRs. Our study showed a direct correlation of the activating 2DS4 receptor with the plasma level of IFN-γ and IL6 in COVID-19 patients, biomarkers that are reported to be elevated during SARS-CoV-2 infection [25] in accordance with our study, although the production of these cytokines is systemic and can be partially attributed to the NK cell production. One recent study reported that the frequency of the 2DS4 gene was significantly increased in COVID-19 patients compared to controls [26].

NK cells play an important role in the innate immune response. Two main populations of these cells are characterized for the absence of lineage-specific markers for T cells CD3, B cells CD19, and monocytes CD14: CD56^bright^ and CD56^dim^CD16^pos^ cells [27]. Nevertheless, among the first population, other two populations can be differentiated: CD56^bright^CD16^neg^ and CD56^bright^CD16^dim^. NK cells that are characterized by cytokine production, including IFN-γ and TNF-α, are an important defense against virus infections. We found a correlation between IFN-γ and CD56^bright^CD16^neg^ NK cells among hospitalized COVID-19 patients. Despite the decreased level of total NK cells observed in these patients compared to convalescents or infection-naïve individuals, the production of IFN-γ was not impaired in patients with moderate COVID-19 in a clinical setting (with no need for ICU admission). The situation of severe COVID-19 is reported to be worse compared to moderate COVID-19 patients or healthy controls [28]. This production of proinflammatory cytokines allows them to promote the activation of inflammation or even autoimmunity [29].

The CD56^dim^CD16^pos^ NK cell subset is characterized by a strong cytotoxicity and high expression of KIR receptors. CD16 binds to the Fc part of the IgG molecules mediating antibody-dependent cellular cytotoxicity (ADCC), allowing the killing of target cells through cytotoxic function (CD56^dim^) or leading to a late inflammatory process (CD56^bright^) [30,31]. In this way, the COVID-19 patients in our study showed a deep depletion of both CD56^dim^CD16^dim^ and CD56^dim^CD16^bright^ NK cell subsets. This depletion was found in convalescents (two months post-infection) only for the CD56^dim^CD16^bright^ NK cell subsets compared to infection-naïve individuals. These two cellular functions, cytotoxicity and cytokine secretion, are further regulated by a balance between the inhibitor and activating receptors [20]. The high frequency of positive subset cells for KIR2DL1 suggests that not only there is a deficient amount of the cytotoxic NK subset, but also that these are impaired by the expression of inhibitory KIR receptors.

Finally, CD56^dim^CD16^neg^ NK cells are unconventional CD56^dim^ cells whose cytotoxic capacity has been found to be lower compared to that of CD56^dim^CD16^pos^ NK cells [32,33]. In this regard, the downregulation of CD16 expression has been also described in Herpes and Varicella zoster infections and might be associated with abnormal NK cell maturation during infection or prolonged cellular activity/activation, driving CD16 shedding, which might be a regulatory mechanism to prevent activation-induced cell death [34,35,36,37]. CD56^dim^CD16^neg^ NK cells are considered to be the precursors of CD56^dim^CD16^pos^ NK cells. In our study, this cell subset was expanded in moderate COVID-19 patients, and the frequency of cells positive for inhibiting 2DL1 and 2DL1/S1 receptors was higher compared to that of convalescents and infection-naïve individuals, which might accentuate the impairment of their cellular functions. This expansion was also found in convalescent individuals two months post-infection, although with less difference compared to infection-naïve individuals. The overexpression of inhibitory 2DL1 in CD56^dim^CD16^neg^ and CD56^dim^CD16^pos^ NK cell subsets was found by Littera et al., who described a higher frequency of the KIR2DL1 and 2DL3 inhibitory KIR gene profile in individuals infected with SARS-CoV-2, suggesting that they are more susceptible to contracting this viral infection, in line with our study [35]. Unfortunately, this study did not differentiate among different NK cell subsets.

On the other hand, the CD56^neg^CD16^bright^ NK cell subset was found to be depleted in COVID-19 patients in our study; this subset is reported to be an unconventional cytotoxic mediator for chronic diseases [15]. NK cells participate in a complex network, interacting with dendritic cells as well as T and B cells through chemokines and a variety of cytokines involved in the differentiation and function of NK cells [23,30,31,38,39].

This study has a number of limitations, including the absence of severe COVID-19 patients and the fact that no longitudinal samples of the patients could have been collected. In addition, functional cell assays and the determination of the KIR genotypes could not be performed in this study due to the limited biological material obtained.

In summary, not only did we find that a different distribution of NK cell subsets in COVID-19 patients could lead to an impaired innate cellular barrier, but we also found that a higher frequency of inhibitory KIR receptors could enhance the impairment of these cells. Another mechanism that can suppress NK cell antiviral activity is the elevated IL-6 and IL-10 levels in COVID-19 through the down-modulation of the activating receptor NKG2D [40,41]. Importantly, this impaired NK cell cytotoxicity is partially recovered earlier after infection. Thus, therapies targeting IL-6 and IL-10 could be investigated as a means for improving NK cell antiviral immunity, especially in severe cases of COVID-19.

## Figures and Tables

**Table 1:** Characteristics of study’s participants at inclusion.

	Hospitalized COVID-19 *n* = 20	Convalescent *n* = 30	Infection Naive *n* = 30
Age (years)	60 (52–72)	45 (31–54)	40 (33–50)
Sex (Female *n*, %)	1 (5%)	18 (60%)	19 (65.5%)
Body Mass Index (Kg/m^2^)	29.1 (24.9–31.1)	23.5 (22.0–24.6)	23.6 (20.8–26.0)
Obesity (>30 BMI)	7 (35%)	1 (3.3%)	1 (3.3%)
Comorbidities (*n*, %)			
Hypertension	7 (35%)	3 (10%)	3 (10%)
Diabetes	6 (30%)	1 (3.5%)	1 (3.5%)
History of positive SARS-CoV-2 RT-PCR	17 (85%) *	19 (63.3%) **	-
History of positive anti-SARS-CoV-2 IgA/IgM/IgG *n*-specific antibodies	20 (100%) *	30 (100%)	0
History of SARS-CoV-2-compatible symptoms	20 (100%) *	19 (63.3%)	0
SARS-CoV-2 IgG S-specific antibodies at inclusion	16 (80%)	30 (100%)	0
Sepsis-related organ failure assessment (SOFA) score			
1	15 (75%)	-	-
2–3	5 (25%)	-	-
Time since SARS-CoV-2 diagnosis (days)	7 (5–9)	60 (26–75)	-

HCW, health care workers; *, at inclusion; **, RT-PCR was performed only in those individuals with SARS-CoV-2 clinical symptoms.

## Data Availability

The original data presented in the study can be seen on request to the corresponding authors.

## References

[1] Wu F., Zhao S., Yu B., Chen Y.-M., Wang W., Song Z.-G., Hu Y., Tao Z.-W., Tian J.-H., Pei Y.-Y. (2020). A new coronavirus associated with human respiratory disease in China. Nature.

[2] Zhu N., Zhang D., Wang W., Li X., Yang B., Song J., Zhao X., Huang B., Shi W., Lu R. (2020). A novel coronavirus from patients with pneumonia in China, 2019. N. Engl. J. Med..

[3] World Health Organization Weekly Epidemiological Update-19 October 2021. https://www.who.int/publications/m/item/weekly-epidemiologicalupdate-19-october-2021.

[4] Guan W.-J., Ni Z.-Y., Hu Y., Liang W.-H., Ou C.-Q., He J.-X., Liu L., Shan H., Lei C.-L., Hui D.S.C. (2020). Clinical characteristics of coronavirus disease 2019 in China. N. Engl. J. Med..

[5] Ferretti A.P., Tomasz K., Yifan W., Nguyen D.M.V., Adam W., Dunlap G.S., Qikai X., Nancy N., Perullo C.R., Cristofaro A.W. (2020). Unbiased screens show CD8 T cells of COVID-19 patients recognize shared epitopes in SARS-CoV-2 that largely reside outside the spike protein. Immunity.

[6] Alba G., Daniela W., Ramirez S.I., Jose M., Dan J.M., RydyznskiModerbacher C., Rawlings S.A., Aaron S., Lakshmanane P., Jadi R.S. (2020). Targets of T cell responses to SARS-CoV-2 coronavirus in humans with COVID-19 disease and unexposed individuals. Cell.

[7] Abel A.M., Yang C., Thakar M.S., Malarkannan S. (2018). Natural killer cells: Development, maturation, and clinical utilization. Front. Immunol..

[8] Andoniou C.E., Andrews D.M., Degli-Esposti M.A. (2006). Natural killer cells in viral infection: More than just killers. Immunol. Rev..

[9] Vivier E., Tomasello E., Baratin M., Walzer T., Ugolini S. (2008). Functions of natural killer cells. Nat. Immunol..

[10] Moretta A., Bottino C., Vitale M., Pende D., Cantoni C., Mingari M.C., Biassoni R., Moretta L. (2001). Activating receptors and coreceptors involved in human natural killer cell mediated cytolysis. Annu. Rev. Immunol..

[11] Fan Y.Y., Yang B.Y., Wu C.Y. (2008). Phenotypically and functionally distinct subsets of natural killer cells in human PBMCs. Cell Biol. Int..

[12] Amand M., Iserentant G., Poli A., Sleiman M., Fievez V., Sanchez I.P., Sauvageot N., Michel T., Aouali N., Janji B. (2017). Human CD56(dim)CD16(dim) cells as an individualized natural killer cell subset. Front. Immunol..

[13] Cao X. (2020). COVID-19: Immunopathology and its implications for therapy. Nat. Rev. Immunol..

[14] Masselli E., Vaccarezza M., Carubbi C., Pozzi G., Presta V., Mirandola P., Vitale M. (2020). NK cells: A double edge sword against SARS-CoV-2. Adv. Biol. Regul..

[15] Forconi C.S., Oduor C.I., Oluoch P.O., Ong’echa J.M., Münz C., Bailey J.A., Moormann A.M. (2020). A new hope for CD56negCD16pos NK cells as unconventional cytotoxic mediators: An adaptation to chronic diseases. Front. Cell Infect. Microbiol..

[16] Soleimanian S., Yaghobi R. (2020). Harnessing memory NK cell to protect against COVID-19. Front. Pharmacol..

[17] Qin C., Zhou L., Hu Z., Zhang S., Yang S., Tao Y., Xie C., Ma K., Shang K., Wang W. (2020). Dysregulation of immune response in patients with Coronavirus 2019 (COVID-19) in Wuhan, China. Clin. Infect. Dis..

[18] Osman M., Faridi R.M., Sligl W., Shabani-Rad M.-T., Dharmani-Khan P., Parker A., Kalra A., Tripathi M.B., Storek J., Tervaert J.W.C. (2020). Impaired natural killer cell counts and cytolytic activity in patients with severe COVID-19. Blood Adv..

[19] Perez-Gomez A., Vitalle J., Gasca-Capote M.C., Gutierrez-Valencia A., Trujillo-Rodriguez M., Serna-Gallego A., Muñoz-Muela E., Jimenez-Leon M.R., Benhnia M.R., Rivas-Jeremias I. (2021). Dendritic cell deficiencies persist seven months after SARS-CoV-2 infection. Cell Mol. Immunol..

[20] Maucourant C., Filipovic I., Ponzetta A., Aleman S., Cornillet M., Hertwig L., Strunz B., Lentini A., Reinius B., Brownlie D. (2020). Natural killer cell immunotypes related to COVID-19 disease severity. Sci. Immunol..

[21] Varchetta S., Mele D., Oliviero B., Mantovani S., Ludovisi S., Cerino A., Bruno R., Castelli A., Mosconi M., Vecchia M. (2021). Unique immunological profile in patients with COVID-19. Cell Mol. Immunol..

[22] Freud A.G., Caligiuri M.A. (2006). Human natural killer cell development. Immunol. Rev..

[23] Leem G., Cheon S., Lee H., Choi J.S., Jeong S., Kim E.S., Jeong H.W., Jeong H., Park S.H., Kim Y.S. (2021). Abnormality in the NK-cell population is prolonged in severe COVID-19 patients. J. Allergy Clin. Immunol..

[24] Stabile H., Nisti P., Morrone S., Pagliara D., Bertaina A., Locatelli F., Santoni A., Gismondi A. (2015). Multifunctional human CD56 low CD16 low natural killer cells are the prominent subset in bone marrow of both healthy pediatric donors and leukemic patients. Haematologica.

[25] Henry B.M., De Oliveira M.H.S., Benoit S., Plebani M., Lippi G. (2020). Hematologic, biochemical and immune biomarker abnormalities associated with severe illness and mortality in coronavirus disease 2019 (COVID-19): A meta-analysis. Clin. Chem. Lab. Med..

[26] Bernal E., Gimeno L., Alcaraz M.J., Quadeer A.A., Moreno M., Martínez-Sánchez M.V., Campillo J.A., Gomez J.M., Pelaez A., García E. (2021). Activating killer-cell immunoglobulin-like receptors are associated with the severity of Coronavirus Disease 2019. J. Infect. Dis..

[27] Dębska-Zielkowska J., Moszkowska G., Zieliński M., Zielińska H., Dukat-Mazurek A., Trzonkowski P., Stefańska K. (2021). KIR receptors as key regulators of NK cells activity in health and disease. Cells.

[28] Ruetsch C., Brglez V., Crémoni M., Zorzi K., Fernandez C., Boyer-Suavet S., Benzaken S., Demonchy E., Risso K., Courjon J. (2021). Functional exhaustion of type I and II interferons production in severe COVID-19 patients. Front. Med..

[29] Osman M.S., van Eeden C., Tervaert J.W.C. (2020). Fatal COVID-19 infections: Is NK cell dysfunction a link with autoimmune HLH?. Autoimmun. Rev..

[30] Ekşioğlu-Demiralp E., Alan S., Sili U., Bakan D., Ocak İ., Yürekli R., Alpay N., Görçin S., Yıldız A. (2021). Peripheral innate and adaptive immune cells during COVID-19: Functional neutrophils, pro-inflammatory monocytes, and half-dead lymphocytes. Cytom. B Clin. Cytom..

[31] Bergantini L., d’Alessandro M., Cameli P., Cavallaro D., Gangi S., Cekorja B., Sestini P., Bargagli E. (2021). NK and T Cell Immunological Signatures in Hospitalized Patients with COVID-19. Cells.

[32] Penack O., Gentilini C., Fischer L., Asemissen A.M., Scheibenbogen C., Thiel E., Uharek L. (2005). CD56dimCD16neg cells are responsible for natural cytotoxicity against tumor targets. Leukemia.

[33] Björkström N.K., Ponzetta A. (2021). Natural killer cells and unconventional T cells in COVID-19. Curr. Opin. Virol..

[34] Romee R., Foley B., Lenvik T., Wang Y., Zhang B., Ankarlo D., Luo X., Cooley S., Verneris M., Walcheck B. (2013). NK cell CD16 surface expression and function is regulated by a disintegrin and metalloprotease-17 (ADAM17). Blood.

[35] Littera R., Chessa L., Deidda S., Angioni G., Campagna M., Lai S., Melis M., Cipri S., Firinu D., Santus S. (2020). Natural killer-cell immunoglobulin-like receptors trigger differences in immune response to SARS-CoV-2 infection. PLoS ONE.

[36] Hsieh W.C., Lai E.Y., Liu Y.T., Wang Y.F., Tzeng Y.S., Cui L., Lai Y.J., Huang H.C., Huang J.H., Ni H.C. (2021). NK cell receptor and ligand composition influences the clearance of SARS-CoV-2. J. Clin. Investig..

[37] Narni-Mancinelli E., Vivier E. (2021). Clues that natural killer cells help to control COVID. Nature.

[38] Mazzoni A., Salvati L., Maggi L., Capone M., Vanni A., Spinicci M., Mencarini J., Caporale R., Peruzzi B., Antonelli A. (2020). Impaired immune cell cytotoxicity in severe COVID-19 is IL-6 dependent. J. Clin. Investig..

[39] Ghasemzadeh M., Ghasemzadeh A., Hosseini E. (2021). Exhausted NK cells and cytokine storms in COVID-19: Whether NK cell therapy could be a therapeutic choice. Hum. Immunol..

[40] Lenart M., Kluczewska A., Szaflarska A., Rutkowska-Zapała M., Wąsik M., Ziemiańska-Pięta A., Kobylarz K., Pituch-Noworolska A., Siedlar M. (2021). Selective downregulation of natural killer activating receptors on NK cells and upregulation of PD-1 expression on T cells in children with severe and/or recurrent Herpes simplex virus infections. Immunobiology.

[41] Campbell T.M., McSharry B.P., Steain M., Ashhurst T.M., Slobedman B., Abendroth A., Mocarski E. (2018). Varicella zoster virus productively infects human natural killer cells and manipulates phenotype. PLoS Pathog..

